# Seed Longevity in Legumes: Deeper Insights Into Mechanisms and Molecular Perspectives

**DOI:** 10.3389/fpls.2022.918206

**Published:** 2022-07-27

**Authors:** Vinita Ramtekey, Susmita Cherukuri, Sunil Kumar, Sripathy Kudekallu V., Seema Sheoran, Udaya Bhaskar K., Bhojaraja Naik K., Sanjay Kumar, Arvind Nath Singh, Harsh Vardhan Singh

**Affiliations:** ^1^ICAR-Indian Institute of Seed Science, Mau, India; ^2^Indian Agricultural Statistics Research Institute-IASRI, New Delhi, India; ^3^ICAR-Indian Institute of Seed Science, Regional Station, Bengaluru, India; ^4^ICAR-Indian Agricultural Research Institute, Regional Station, Karnal, India; ^5^ICAR-National Bureau of Agriculturally Important Microorganism, Mau, India

**Keywords:** seed longevity, legumes, molecular pathways, genes, crop productivity

## Abstract

Sustainable agricultural production largely depends upon the viability and longevity of high-quality seeds during storage. Legumes are considered as rich source of dietary protein that helps to ensure nutritional security, but associated with poor seed longevity that hinders their performance and productivity in farmer's fields. Seed longevity is the key determinant to assure proper seed plant value and crop yield. Thus, maintenance of seed longevity during storage is of prime concern and a pre-requisite for enhancing crop productivity of legumes. Seed longevity is significantly correlated with other seed quality parameters such as germination, vigor, viability and seed coat permeability that affect crop growth and development, consequently distressing crop yield. Therefore, information on genetic basis and regulatory networks associated with seed longevity, as well as molecular dissection of traits linked to longevity could help in developing crop varieties with good storability. Keeping this in view, the present review focuses towards highlighting the molecular basis of seed longevity, with special emphasis on candidate genes and proteins associated with seed longevity and their interplay with other quality parameters. Further, an attempt was made to provide information on 3D structures of various genetic loci (genes/proteins) associated to seed longevity that could facilitate in understanding the interactions taking place within the seed at molecular level. This review compiles and provides information on genetic and genomic approaches for the identification of molecular pathways and key players involved in the maintenance of seed longevity in legumes, in a holistic manner. Finally, a hypothetical fast-forward breeding pipeline has been provided, that could assist the breeders to successfully develop varieties with improved seed longevity in legumes.

## Introduction

Seed longevity is a mere expression of retaining the germination ability during the process of dry storage. It is a complex phenomenon governed by several intrinsic as well as extrinsic factors to which seeds are exposed during maturity and storage. In cultivated crop species, greater the longevity means greater the chance for meeting the norms of regulatory regime with desirable germination, thereby contributing to crop production and adding economic value in the system. Understanding the mechanism of seed longevity has greater ecological, agronomical and economical significance. Legumes belonging to the family of Fabaceae are nutritionally invaluable, providing proteins with essential amino acids together with carbohydrates, fibers and vitamins (Bailly, 2004; Bouchenak and Lamri-Senhadji, 2013; Annor et al., 2014), however associated with the problem of poor seed longevity. Seed longevity in legumes greatly varies within the species, and can be ascertained based on the P_50_ value (half viability index). Nagel and Borner (2010) reported that longevity was highest in pea, followed by lupin, common vetch and beans among the referred legumes. However, varied intrinsic and extrinsic determinants have a say in exact seed longevity durations and legume seeds, where oil is major form of storage exhibit shorter P_50_ value. In legumes like soybean, the interference of maternal tissues (Haughn and Chaudhury, 2005) and cytoplasmic gene action has been reported, that infer the complexity of systems involved in longevity *per se* (Debeaujon et al., 2000; Hundertmark et al., 2011; Nagel et al., 2015). Pioneering studies of Ellis and Roberts (1980) gave insights on abiotic factors *viz*. humidity, temperature and oxygen concentration. Along with them, genetic and pre-storage factors were also given utmost priority in understanding seed longevity mechanisms (Ellis and Roberts, 1980; Ellis et al., 1989).

In majority of crop species longevity is expressed with onset of physiological maturity and same was studied in legumes like soybean. Even though onset of germination is much prior in some of the species, progressive increment in longevity at the end of seed filling after the attainment of desiccation tolerance was documented. At this juncture, progressive increase in longevity can be attributed to gene expression of encoding chaperones like heat shock proteins (HSPs) and late embryogenesis abundant (LEA) proteins (Hundertmark et al., 2011; Chatelain et al., 2012; Sano et al., 2016), increase of non-reducing sugars and raffinose family oligosaccharides (RFOs) (Rajjou and Debeaujon, 2008; Waterworth et al., 2010). Decrease in metabolic activities and onset of glassy state (Salvi et al., 2016; Leprince et al., 2017) in living cells of seed counteracts deterioration changes and factors in seed longevity improvement. Role of secondary metabolites like tocopherols, flavonoids and glutathione were also thoroughly explained in various studies (El-Maarouf-Bouteau, 2022). Oxidation of biomolecules *viz*. proteins, lipids and nucleic acids by reactive oxygen species (ROS) mediated mechanisms, and repair activity (Tejedor-Cano et al., 2010; Nagel et al., 2015) that counteract the deterioration changes, could also determine the extent of seed longevity in diverse plant species. To reap desired dividends of improved crop varieties, harvesting should be at a stage where longevity is at its maximum, however risks pertinent to field weathering, shattering, seed handling bottlenecks should be factored in the decision-making process. Moreover, seed dormancy is also one such enigmatic mechanism that plays a protective role in longevity *per se* and its expression depends on intrinsic hormonal balance and external abiotic situations. The molecular control of both preventive and curative aspects was accounted by many researchers, current review is an attempt in assembling the studies on legume species within the realm of seed longevity. The review focuses on providing information on the molecular basis of genetic factors that are directly and/or indirectly *via* other seed quality parameters, involved in modulating the inter-connected regulatory networks of seed longevity in legumes.

## Harmony Amid Seed Longevity With Other Seed Quality Parameters

**Figure 1:**
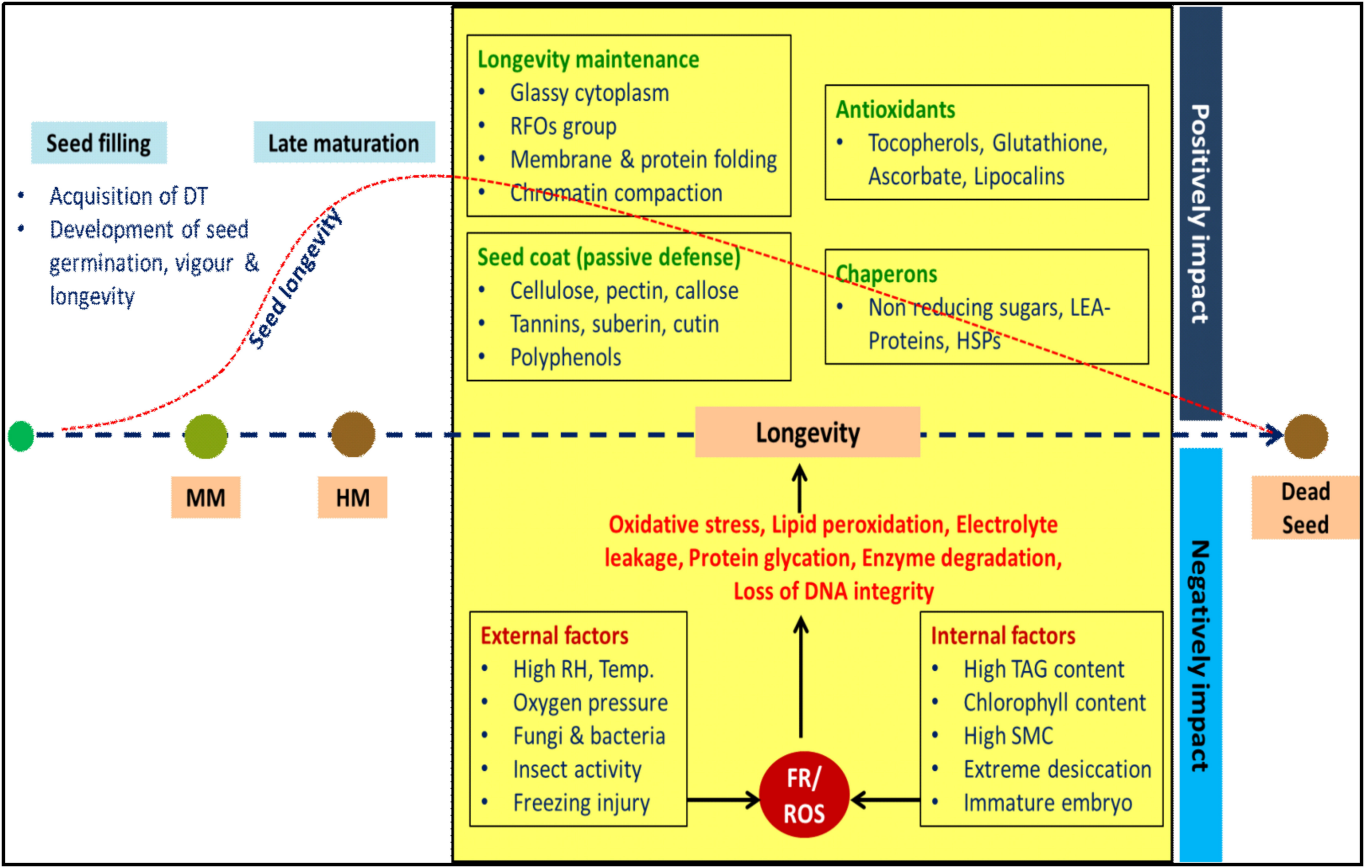
Schematic representation of interplay between factors determining seed longevity. Seed attains maximum dry weight during mass maturity (MM). Seed vigour and longevity progressively developed during late maturation phase and reaches maximum during harvest maturity (HM). During dry storage, longevity is a result of interaction between various intrinsic and extrinsic factors. Seed longevity is not indefinite; it decreases gradually and lost completely at some point of time. DT: desiccation tolerance; RFOs: raffinose family oligosaccharides; LEA: late embryogenesis abundant; HSPs: heat shock proteins, RH: relative humidity, TAG: triacylglycerol; SMC: seed moisture content, FR: free radicals, ROS: reactive oxygen species.

In plants, fascinating set of events taking place during post-fertilization results in the development of a dispersal unit known as seed. Seeds are nature's gift for enhanced crop production and means for ensuring food security. During domestication, commercialization and improvement of crops seed, seed quality is defined as a measure of characters or attributes, which will determine the performance of seed when it is sown or stored (Hampton and Hill, 2002). After harvest, mostly seeds were stored before use, and the storage period may be relatively short (few weeks or months) or long (for few years). Seeds age during storage, resulting in decline in quality and ultimately loss of viability (breakdown of metabolic system), if storage conditions are not conducive (Harrington, 1972). The term seed longevity refers to a period that a seed population maintains viability under certain environmental conditions (Ellis, 1991), which is often directly related to seed vigor. High vigor seeds ensure uniform emergence and plant stand establishment under wide range of environmental conditions, and retain these traits even after storage longevity (Finch-Savage and Bassel, 2016; Hay et al., 2019). Poor longevity negatively impacts seedling establishment and consequently crop yield. Seed longevity *per se* is a trait of interest not only for breeding programmes where seed persistence in storage is a problem (oilseed and pulse crops), but also for describing natural population dynamics over time and space (Finch-Savage and Bassel, 2016). Survival of seed in the dry state is a cause and effect of several molecular and cellular processes that occur during seed development, maturation and post-harvest storage conditions. The interaction of seed longevity with other quality parameters is presented in Figure 1.

### Intrinsic Factors Determining Seed Longevity

Genetic makeup of seed (chemical composition and genes governing lifespan) explicitly determines inherent storage potential. Seeds with low lipid content (<10%) have better longevity compared to seeds with high lipid content. Similar to this, seeds that retain chlorophyll (chlorophyllous seeds) tend to have poor longevity (Ballesteros et al., 2020). Embryo interaction with external environment is overseen by maternally derived seed coat or pericarp, thus its structure and composition are critical for maintenance of seed longevity. Presence of complex polysaccharides (cellulose, pectin, and callose), tannins, suberin, cutin, polyphenols impart physical and chemical resistance to seed coat, which is key for longevity (Sano et al., 2016). Genes regulating synthesis of HSPs, LEA-proteins, antioxidants are vital for the prolonged persistence of seed. The water content in the seed is one of the most important factors affecting the seed longevity, free water in cytoplasm will increase respiratory rate resulting in seed deterioration mediated by ROS (Kumar et al., 2015). Similarly, in over-dried seeds, extreme dehydration disrupts the interface of water film covering the cellular structure and exposes macromolecules of membranes, making them vulnerable to ROS (Mira et al., 2019). Hence, both high and extremely low seed moisture accelerates ROS-mediated lipid peroxidation leading to faster seed aging. The longevity of endospermic seeds stored at 45°C and 60% relative humidity (RH) were 3.3-fold lower than the non-endospermic seeds, signifying the effect of maternal genome on seed storability (Probert et al., 2009). Plant hormones such as abscisic acid (ABA) and auxins significantly influence longevity by regulating many genes encoding LEA-proteins and RFO synthesis (Zinsmeister et al., 2020).

Developing seed, acquires seed quality sequentially during seed development and maturation. Even though seed attains maximum dry weight during mass maturity, longevity is acquired gradually during late maturation phase and 30–50-fold increase in longevity has been noticed across various species (Verdier et al., 2013; Hay et al., 2015; Righetti et al., 2015). The relative length of late maturation phase varies across the species, ranging from 45% of total seed developmental time in soybean, 30% in French bean, and 78% in rice (Leprince et al., 2017). Hence, thorough understanding of seed maturity is crucial for obtaining seeds of highest quality. Harvesting the crop before it attains complete maturity often results in inadequate development of essential structures in seed, that may result in low seed yield and poor longevity (Ekpong and Sukprakarn, 2008; Wang et al., 2008). Similarly, harvesting too late may increase the risk of shattering and may decrease the quality of seed due to aging.

### Extrinsic Factors Determining Seed Longevity

During seed development, changes in environmental factors (temperature, photoperiod, nutrition, and water availability), damage caused by pests and diseases, and mechanical damage during harvesting, transportation, directly affect seed longevity. The consequences of above events will be in the form of disabled enzymatic functions and subcellular structures resulting in decreased quality and shorter lifespan (Sano et al., 2016). During storage, seed longevity hinges on RH, temperature, oxygen pressure, and activity of microflora and insect pests (Copeland and McDonald, 2001; Schwember and Bradford, 2010).

Storage temperature affects seed longevity primarily by altering the activity of the enzymes in the seed. Higher the storage temperature and seed moisture content, stronger the seed metabolism and vice-versa (Copeland and McDonald, 2001). Hence, reasonable control over these factors is required to improve seed longevity during storage. ROS oxidize or per-oxidize several macromolecules such as lipids, nucleic acids and proteins, causing damage to sub-cellular structures resulting in reduction of seed viability and vigor (Kumar et al., 2015; Fleming et al., 2019). An increased oxygen level during storage was positively correlated to more chromosomal aberrations during cell division (Abdalla and Roberts, 1968). Storing *Vicia faba* seeds for 4 weeks at oxygen pressures of 3 or 6 mega pascals (MPa) resulted in increased chromosomal defects (Moutschen-Dahmen et al., 1959). The ultra-dried stored seeds of brassica survived for 36 years when stored in glass vials filled with modified atmosphere (CO_2_ enriched) (Gonzalez-Benito et al., 2011). Similarly, the storage of soybean at elevated oxygen pressure (0.77 MPa) at 25°C and 17% moisture content (MC) resulted in loss of seed germination within 3 weeks, whereas the decline was not observed in seeds stored below 0.77 MPa nitrogen (Ohlrogge and Kernan, 1982). These studies clearly portray that reduced oxygen levels extend seed longevity during storage.

In soybean, water stress during seed development result in mature green seeds with reduced seed longevity (Smolikova et al., 2021). Contrastingly, a water stress in groundnut during seed maturation can enhance seed longevity (Ramamoorthy and Basu, 1996). The differential response of longevity to moisture stress in above species may be because of the ability of species to modulate the maturation process to regain lost longevity to some extent. Soil nutrition and status of macro and micro elements in mother plant affects seed yield and also longevity. Low nitrogen supply affects protein synthesis and cell wall metabolism (He et al., 2014), resulting in poor seed longevity. In barley, plants cultivated with adequate nutrient supply produced seeds with better longevity, in comparison to the plants grown under nutrient deficit stress conditions.

### Physiological and Bio-Chemical Events Underlying Acquisition of Desiccation Tolerance and Seed Longevity

After completion of series of cell division and cell differentiation, seed development switches to the maturation phase that can be divided into early (reserve accumulation) and late maturation (maturation drying). Desiccation tolerance is defined as the ability of living entity to deal with extreme moisture loss to levels below 0.1 g H_2_O per gram DW (dry weight) or drying to RH below 50% and subsequent re-hydration without accumulation of lethal damage (Alpert, 2005; Leprince and Buitink, 2010). During early seed maturation, embryo accumulates specific molecules that are associated with the cells ability to tolerate extreme water stress *viz.*, low molecular weight antioxidants, oligosaccharides such as raffinose, stachyose, LEA-proteins, and HSPs. Large amounts of non-reducing carbohydrates (sucrose, raffinose, and stachyose) are responsible for induction of structural stability to membranes and proteins by replacing of water molecules as a strategy to survive desiccation. Whereas accumulation of reducing sugars (fructose, glucose, and xylose) is detrimental to seed longevity due to enhanced glycation of proteins (Leonova et al., 2020). In legumes, among non-reducing sugars, accumulation of high molecular weight RFOs (raffinose and stachyose) occurs in higher proportions as compared to sucrose. Higher the molecular weight, higher the glass transition temperature (Tg) and early glass formation during drying (Leprince et al., 2017). During late maturation, the reducing sugars disappears progressively and proportion of non-reducing sugars increase in the cytoplasm (Verdier et al., 2013). At the end of late maturation phase, transition of liquid crystalline state of membranes to gel phase is achieved.

Based on the desiccation tolerance, seeds are classified into three types *viz.*, orthodox (tolerate drying up to 5% MC and storage under subzero temperatures), recalcitrant (intolerant to drying and low temperature storage), and intermediate (tolerate some degree of drying, but sensitive to low temperature storage; Roberts, 1973; Azarkovich, 2020). The maturation in orthodox seeds is accompanied with a water loss up to 5–10% weight by weight (w/w), which allows them to sustain unfavorable environmental conditions, such as extremely high and low temperatures and drought. As detailed above, the tolerance capacity in the orthodox seeds is due to restricted metabolism (following drying) and strengthened antioxidant system, that is dependent on a complex signaling network (Berjak and Pammenter, 2013; Ratajczak et al., 2018). Along with this, accumulation of desired levels of soluble sugars, mainly the RFOs (Leonova et al., 2020) and the presence of LEA proteins and HSPs confer desiccation tolerance in orthodox seeds (Hundertmark et al., 2011; Chatelain et al., 2012; Sano et al., 2016). Added to this, the lifespan of orthodox seeds can be prolonged when they enter the vitreous state (Walters et al., 2005; Buitink and Leprince, 2008), wherein the cellular metabolic processes remain minimal or arrested completely (Kranner et al., 2010). In orthodox seeds, the mechanisms behind the onset of desiccation tolerance are activated at the early stages of maturation (Leprince et al., 2017). Later on, desiccation tolerance is lost during germination, at the moment of radicle emergence (Smolikova et al., 2021). To a large extent, cultivated legume species show similar pattern of orthodox seed behavior and variations in seed longevity do exist among the species. Contrastingly, in recalcitrant seeds, desiccation leads to damage and loss of viability. Accumulation of ROS serves as the principal driving force behind loss of viability in the seeds of recalcitrant and intermediate behavior during drying and storage (Kurek et al., 2019). Loss of balance between ROS production and scavenging occurs due to the low activity of antioxidant system and differences in the content of LEA proteins (Berjak and Pammenter, 2013). Apart from this, sensitivity of these seeds to desiccation is further increased by their structure, which does not protect them against mechanical damage during stress conditions (Berjak and Pammenter, 2008).

Late seed maturation is accompanied by degradation of chlorophyll and carotenoids (Teixeira et al., 2016). Retention of chlorophyll in achlorophyllous seeds indicates immaturity, and is detrimental for seed longevity (Jalink et al., 1998). Whereas, catabolism of chlorophyll pigment releases phytyl chain, which serve as substrate for tocopherol synthesis (antioxidant involved in seed longevity) (Durrett and Welti, 2021). Similarly, HSPs gathered during late maturation phase act as chaperones and aid in imparting stabilization to proteins during folding (Kotak et al., 2007). Likewise, accumulated antioxidants, such as tocopherols, glutathione, ascorbate, polyols, quinones, and secondary metabolites such as flavonoids in testa of seed coat are known to affect seed longevity (Sano et al., 2016). In legumes, deposition of substances such as cutin, suberin, tannins, lignin, pectin, and β-glucans in testa act as a physical barrier and offer passive defense against pathogen infection and mechanical stress. Further, certain structural changes occur at cellular level during maturation phase, such as folding of cell wall and dismantling of thylakoids in chloroplasts (Nagel et al., 2015; Ballesteros et al., 2020). The constituents of cytosol are compressed during dehydration that is accompanied by increased viscosity of cytoplasm, reduced diffusion of water and oxygen and dramatic reduction of cellular responses in seeds (Buitink and Leprince, 2008). The resultant structure is greatly disorganized and comprises of pores having different shapes and sizes and hence, dehydrated cytoplasm is known as glassy matrix (Ballesteros et al., 2020).

LEA-proteins consist of high level of glycine contents, low amount of cysteine and tryptophan residues, and high proportion of alanine, glutamate, lysine, arginine, and threonine (Battaglia et al., 2008). Due to their primary nature, LEA-proteins remain stable under a broad range of temperatures. During cell dehydration, LEA-proteins also act as chaperones and are involved in structural stabilization of denatured proteins and promote their refolding through intensive hydrogen bond formation (Smolikova et al., 2021). LEA-proteins are also responsible for sequestration of ionic compounds that accumulate during cell dehydration, and protection of membrane proteins and enzymes from the deleterious effects of increased salt concentration (Conde et al., 2011). Other structural adaptations that occur during this stage are chromatin compaction and nuclear size reduction, which are reversed during germination (van Zanten et al., 2011). All these physio-chemical changes that occur in a developing seed (more distinctly during late maturation phase) cumulatively contribute to maximization of seed vigor and longevity.

### Variations in Seed Longevity of Orthodox Seeds: Bio-Physical Perspective

Even though state of quiescence is achieved in seed, when water content falls below 0.1 g H_2_O per gram DW (cytoplasm transform into glassy matrix), at the end of maturation phase seed cannot escape deterioration. Under optimum conditions of storage, seed biochemical composition and cell structures determine the longevity (Ballesteros et al., 2020). For instance, pea seeds that lack storage lipids and chlorophyll have better longevity whereas, poor longevity in groundnut is due to presence of higher proportion of lipid bodies (Fleming et al., 2019).

Ballesteros et al. (2020) hypothesized different aging mechanisms based upon structural conformations in dry desiccation tolerant cells. The biophysical perspective of seed longevity as a result of interplay between chlorophyll content and storage lipids is being discussed below. During seed filling stage, embryo accumulates high amounts of starch, protein or lipids, replacing over 50% of cellular water (Walters, 2015) and at late maturation phase, photosynthetic pigments get degraded (Leprince et al., 2017). Characteristically, in achlorophyllous cells with low lipid content as observed in majority of cereals and pulses, favor accumulation of copious complex carbohydrates, proteins and lipid constitute <10% of total dry matter accumulation. Largely accumulated proteins and starch along with other cell constituents especially antioxidants serve as scavengers of ROS or reduce the mobility of oxidized lipid fragments within the glassy matrix (Kumar et al., 2021). Sparsely dispersed small lipid bodies act as superior scavengers of ROS in the glassy matrix that attribute for better seed longevity in majority of cereals and pulses (Zinsmeister et al., 2020). In species of *Medicago truncatula*, that retain chlorophyll after completion of maturation along with low lipid content in cells, relatively poor seed longevity has been noticed (Rajjou and Debeaujon, 2008). Persistent chlorophyll in cells often tends to get photo-excited, and release free electrons which can oxidize macromolecules even in the absence of free water molecules. Oilseed crops such as soybean and groundnut typically possess achlorophyllous cells with high amount of lipid bodies (upto 40% of total dry matter). Generally, presence of free fatty acids is linked to poor seed longevity. ROS easily per-oxidize lipid bodies resulting in extensive cell damage during storage (Kumar et al., 2015). During maturation drying, water soluble cellular constituents shrink and solidify, whereas lipid molecules remain fluid, and get crystallized when subjected to freezing temperatures (Mira et al., 2019). As lipid bodies crystallize, they shrink and pore space between them and solidified cytoplasm increases, potentially disrupting the glassy matrix leading to poor storability.

## Molecular Pathways Underlying Seed Longevity

Understanding the molecular mechanisms underlying seed development is crucial to decipher the genes and metabolic pathways involved in its regulation from embryo to maturation, as well as transitioning of the dormancy state to germination. The application of genotyping and omics tools has improved our understanding about genes, associated RNAs and proteins that are involved in the metabolic pathways of seed development (Li and Li, 2016). Seed longevity being an important trait varies largely based on plant species, genotype and environmental conditions (Joosen et al., 2012), hence explicating the molecular mechanisms underlying seed longevity remains complicated. In legumes seed longevity is acquired gradually throughout seed maturity from seed filling onwards (Verdier et al., 2013). The processes governing seed longevity and vigor acquisition during late maturity are yet unknown, whereas the mechanisms involved in regulating longevity during storage are well-studied. The RNAseq results and co-expression network analysis demonstrated that complex transcriptome alterations occur during physiological maturity until the dry state, revealing multiple transcription factors (TFs) could be linked in expanding seed lifetime. In *M. truncatula* and Arabidopsis, some of these TFs were previously found in a gene co-expression network linked to longevity (Righetti et al., 2015). It suggests that a post-abscission pathway is being activated to prepare seed for the dry state and germination, by manufacturing mRNA that will be preserved until seed imbibition (Nakabayashi et al., 2005). This hypothesis is supported by a number of observations. For example, in *M. truncatula*, several homologs for these TFs were discovered to be co-regulated with the induction of longevity (Verdier et al., 2013). The majority of TFs were reported to be involved in germination and growth. Some of them include homolog of WRKY6, that acts as a positive regulator of ABA signaling during seed germination and early seedling development in Arabidopsis (Huang et al., 2016), a homolog of SOMNUS, a homolog of HOMEOBOX-1 (HB-1) both involved in hypocotyl growth under short photoperiods (Capella et al., 2015), and a homolog of INDETERMINATE DOMAIN1/ENHY helps in the transition of seed germination by regulating gibberellic acid (GA) and ABA signaling during seed maturation (Feurtado et al., 2011). During late seed maturation, accumulation of transcripts associated with protein degradation *via* SCF family of modular E3 ubiquitin pathway increased. This suggests that before entering the dry state, seeds establish a machinery for post-translational regulation, which is anticipated to get activated upon imbibition.

**Figure 2:**
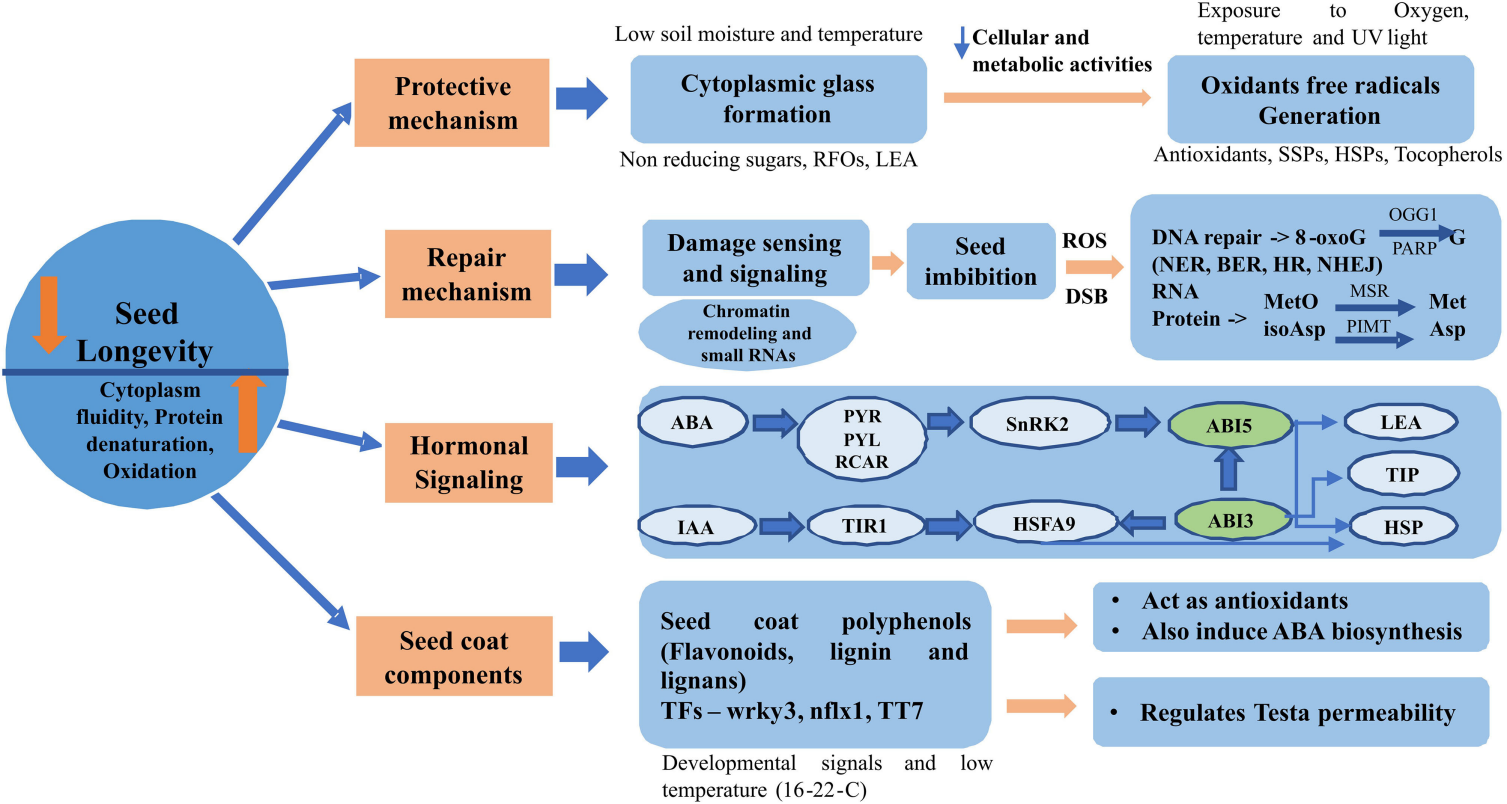
Molecular mechanisms involved in regulating seed longevity during storage. ABA: abscisic acid, BER: base excision repair, DSB: double strand breaks, HR: homologous repair, SSP: seed storage proteins, HSP: heat shock protein, IAA: indole-3-acetic acid, LEA: late embryogenesis abundant, MSR: methionine sulfoxide reductase, NHEJ: non-homologous end joining, NER: nucleotide excision repair, PIMT: protein L -isoaspartyl methyltransferase, PYR/PYL/RCAR: pyrabactin resistance/PYR1-like/regulatory components of ABA receptors, RFOs: raffinose family oligosaccharides, ROS: reactive oxygen species, snRK2: sucrose non-fermenting 1- related subfamily 2, TIP: tonoplast intrinsic protein, TIR1: transport inhibitor response 1, TF: transcription factor.

Recent research evidences suggest four molecular mechanisms based on protection, repair, hormonal signaling and *via* seed components to be mainly involved in regulating seed longevity during seed storage (Figure 2).

### Protective Mechanisms

Different classes of seeds act contrarily under dehydration and storage, such as orthodox seeds endure desiccation at maturation and uphold their germination capacity for longer periods by owning mechanisms that maintain metabolic quiescence. While recalcitrant seeds undergo slight or no drying during maturation and go on desiccation sensitive due to which it can't survive during *ex-situ* conservation (Rajjou et al., 2012). The seed ability to endure desiccation is an indispensable trait to enhance longevity and vigor, and ensure successful germination. Under dehydration, recalcitrant seeds couldn't preserve DNA integrity while orthodox seeds stimulate their protective mechanisms that help in procurement and maintenance of desiccation tolerance (Buitnik et al., 2002; Weitbrecht et al., 2011). In dehydrated state the presence of antioxidants, accretion of molecules wielding protective functions, e.g., sucrose and other oligosaccharides, LEA proteins, disposition of amphiphatic molecules and the existence of repair mechanisms during rehydration helps to maintain the seed longevity in desiccation tolerant seed (Ventura et al., 2012). Many researchers opined that, in legumes such as soybean, seed longevity is obtained during seed maturation from the phenological stage onwards i.e., a few days after achieving desiccation tolerance and immediately before the completion of seed filling and at the start of maturation drying. After that, longevity doubles till stage R9, which corresponds to dry mature seeds (Lima et al., 2017). There is growing evidence that the synthesis of defensive mechanisms that help seeds to live longer is triggered during these stages and increase in intensity, as the seed matures (Probert et al., 2007; Basso et al., 2018). LEA genes are increased during seed filling in the legume *Medicago truncatula*, but a specific group of LEA polypeptides accumulate afterwards in association with longevity (Chatelain et al., 2012). Lima et al. (2017) observed increased levels of transcripts encoding HSPs and heat shock factors (HSFs), as well as increased concentration of RFOs, which both contribute to soybean resistance against accelerated aging (Tejedor-Cano et al., 2010). As a result, soybean seeds harvested at different stages of maturity are likely to have varying protective molecules.

### Repair Mechanisms

Seed viability depends on maintaining the integrity of DNA, RNA and protein molecules (Priestley, 1986) which are susceptible to spontaneous degradation because of aging. The long dry quiescent period followed by desiccation and rehydration cycle decrease the cell's maintenance activities and lead to accumulation of cellular damage to these components (Powell and Matthews, 2012). During these stages, scavenging of excess ROS is very critical for seed longevity, as it accelerates the seed aging by causing oxidative stress and DNA strands breaks either through denaturation or base modifications (Kranner et al., 2010; Zhou et al., 2020). The repair activities reverse impairment to molecules, reinstating normal cellular function. Genetic studies have revealed the importance of the cellular repair pathways to maintain viability of the quiescent seed (Waterworth et al., 2015). During germination, seed imbibes water and rapidly activate processes like respiration, *de novo* protein synthesis, ribosomal and messenger RNA production along with mitochondrial ATP synthesis. Upon seed imbibition, the repair mechanism recovers the damaged cellular structures during quiescence depending on the genotype (Powell and Matthews, 2012).

#### DNA Repair

The accumulation of macromolecular damage, such as DNA damage and genomic instability, is thought to be one of the driving forces in the aging process (Bertram and Hass, 2008). The DNA damages accumulate in embryo by base loss, base modifications, single-strand DNA breaks (SSBs), double-strand DNA breaks (DSBs) and generating abasic sites (Cordoba-Canero et al., 2014) need to be repaired early, to maintain the germination potential (Waterworth et al., 2016). Seed industries are attempting to induce repair mechanism during invigoration treatments of seeds, which are based on controlled hydration of the seeds referred to as priming. DNA repair has been demonstrated to occur during seed priming (Huang et al., 2008). Cellular endurance depends on the repair pathways for base damage *via* base excision repair (BER), whereas SSBs are repaired by nucleotide excision repair (NER) and DSBs are repaired by homologous repair (HR) or non-homologous end joining (NHEJ) mechanisms. Several DNA ligases have also played explicit roles in the NHEJ pathway of DSBs repair as well as SSBs in Arabidopsis to maintain seed quality and longevity (Ellenberger and Tomkinson, 2008; Andreuzza et al., 2010; Waterworth et al., 2010). Proteins involved in seed DNA repair mechanisms, on the other hand, are still poorly understood. DNA in seeds is exposed to a very different chemical environment than DNA in the nucleus of actively metabolizing cells. Not surprisingly, the activity of poly (ADP-ribose) polymerases (enzymes involved in DNA base-excision repair, DNA-damage signaling, and genomic stability regulation) have been shown to be required for early germination (Hunt et al., 2007). Maintaining a functional DNA repair complex appears to be a requirement for long-term survival in the dry state. Cell damage can also happen when chromatin or DNA forms are not well-structured to allow water loss during dehydration. The chromatin remodeling by mono-ubiquitination and protein glycation helps to regulate ABA levels in seeds, playing a critical role in dormancy (Liu et al., 2007) and enabling the transition to a compatible DNA configuration under a dehydrated state (Talasz et al., 2002).

Maintaining DNA integrity during seed storage is important for seed longevity (Zhou et al., 2020; Raquid et al., 2021). Among 11 genes with the ontology of DNA damage/repair, a dominant-negative mutant of KIN14h, an Arabidopsis gene encoding kinesin-like protein 1 (KP1) that regulates seed respiration, showed increased germination rate (Yang et al., 2011). A null mutant of *Os*ALDH7, which encodes aldehyde dehydrogenase 7, showed reduction in germination rate after accelerated aging in rice (Shin et al., 2009), indicating its role as a potential candidate gene for seed longevity. Several other genes such as tyrosyl-DNA phosphodiesterase 1 (TDP1) and transcription elongation factor II-S (*Mt*TFIIS) are also found in *M. truncatula* regulating seed longevity (Macovei et al., 2011).

#### RNA Repair

Owing to its single-stranded structure, RNA is more vulnerable to ROS oxidation than DNA. The loss of germination ability was found to be accompanied by a decrease in total RNA content and RNA integrity (Kranner et al., 2011). Damaged mRNA prevents translation, and a decrease in translational activity in imbibed seeds resulting in decrease of seed longevity (Rajjou and Debeaujon, 2008). During the seed maturation phase, mRNAs required for germination were found to be accumulated in Arabidopsis and rice (Sano et al., 2015). It has been proposed that the early translation of stored mRNAs allows seeds to resume metabolic activity quickly after imbibition (Sano et al., 2015). The stored mRNAs originated from genes with ABA-responsive promoters, advocates that ABA-mediated transcription plays a vital role in determining stored mRNAs profiles (Kimura and Nambara, 2010). They ascend from the stress tolerance response genes and proteins such as LEA genes and HSPs. Lately small non-coding RNAs (miRNAs and siRNAs) have also been projected to play significant role in DSB repair. In this context, additional research should be carried out to unravel the clear-cut role of small RNA networks in seed longevity.

#### Protein Repair

Proteins present in seeds also endure a varied range of post-translational modifications such as carbonylation, nitrosylation that regulate their action, solidity and subcellular localization (Job et al., 2005; Bai et al., 2011). Protein synthesis is an essential requirement for seed germination (Rajjou et al., 2004). When compared to the *de novo* protein synthesis, protein repair is quite inexpensive in terms of energy requirements to restore functional activity of damaged proteins. This strategy appears to prevail in context of dry quiescent seeds in order to initiate seed germination and restart metabolism. *De novo* protein synthesis from stored mRNA can enable the renewal of non-functional proteins that were altered during storage and are required to restart metabolism during germination (Rajjou and Debeaujon, 2008). According to the previous findings, the germination process increases the synthesis of several enzymes involved in methionine metabolism, such as methionine synthase, S-adenosyl methionine synthetase, and S-adenosyl homocysteine hydrolase (Rajjou et al., 2006). Likewise, the cellular activity in germinating seeds is reactivated due to the methionine and S-adenosyl methionine (AdoMet) role in plant metabolism (Gallardo et al., 2002). Another possibility is that germinating seeds have a special need for methionine and/or AdoMet. The plant protein L-isoaspartyl methyl transferases (PIMT), an AdoMet-dependent enzyme changes anomalous L-isoaspartyl residues (isoAsp) to their typical L-aspartyl forms (Asp). It has been depicted in rice and chickpea that the overexpression of PIMT proteins in seeds enhanced seed vigor and longevity (Verma et al., 2013; Petla et al., 2016). Indeed, naturally aged barley seeds have lower levels of PIMT activity, whereas sacred lotus seeds, which hold the world record for long-term seed viability of 1,300 years, have high levels of this enzyme during germination (Rajjou and Debeaujon, 2008). The highest PIMT activity has been found in plants, primarily in seeds, where non-enzymatic protein damage is thought to occur during dehydration and dry storage (Mudgett et al., 1997). As mentioned above, for DNA repair the maintenance of a functional protein repair mechanism appears to be a key condition for long-term survival of seeds in the dry state.

### Hormonal Signaling

Both seed longevity and dormancy are determined by maternal genotype and controlled by ABA signaling (Sano et al., 2016). According to mutational studies, abscisic acid insensitive 5 (ABI5) plays a role in seed longevity regulation by controlling RFO and LEA protein levels, as well as the expression of photosynthesis-related nuclear genes. Other legume crops, such as chickpea, pea, and soybean have also been studied to determine the importance of RFO levels and the expression of genes such as ABI5, raffinose synthase, and galactinol synthase in controlling seed longevity and vigor during seed maturation and germination (Zinsmeister et al., 2016; Lima et al., 2017). Abscisic acid insensitive 3 (ABI3) has been linked to seed longevity signaling *via* the seed-specific heat shock factor HSFA9 (Sano et al., 2016), as reported in Arabidopsis and sunflower (Prieto-Dapena et al., 2006; Kotak et al., 2007). Along with ABA, ethylene and auxins were also deciphered to play a significant role in determining seed longevity. For instance, simultaneous heterologous expression of the drought responsive element binding (DREB) factor in sunflower i.e., *Ha*DREB2 of the apetala2/ethylene response binding protein (AP2/ERBP) family, resulted in enhancement of longevity in seeds (Almoguera et al., 2009; Shu et al., 2018). Similarly, longevity genes were discovered in *M. truncatula* that were enriched with auxin-binding factor binding sites (Righetti et al., 2015), indicating the probable role of auxins in regulating seed longevity.

### Seed Components

The maternal tissue of the seed coat i.e., testa, functions as a protective barrier between the embryo and the external environment, modulating seed germination, and dormancy (Haughn and Chaudhury, 2005). Polyphenols, polysaccharides, suberin, cutin, and other macromolecules found in seed coat, confer physio-chemical resistance to cells. Several polyphenols such as flavonoids, lignins, and lignans were discovered to be accumulated in vacuoles during early seed development that get oxidized during seed desiccation (Pourcel et al., 2005). Several studies have found that these polyphenols could have an effect on longevity (Debeaujon et al., 2000; Zhang et al., 2006). Flavonoids act as antioxidants and scavenge excess ROS, thus limiting the oxidative stress. Pro-anthocyanidins (PAs), also known as condensed tannins and one of the predominant forms of flavonoids, have been shown to induce ABA biosynthesis in Arabidopsis seeds (Jia et al., 2012), potentially influencing seed longevity.

The testa flavonoids are accumulated solely on developmental signals (Debeaujon et al., 2000), but a recent study suggests that their biosynthesis may be tempered by environmental factors such as more PAs accumulated in seed coat when maternal plants are grown at low temperatures (16–22°C) (MacGregor et al., 2015). The seed coat's antimicrobial quinones and insoluble polymers act as a barrier to water and oxygen pervasion, mechanical impairment, and enhance biotic as well as abiotic stress resistance (Pourcel et al., 2005). A number of defensive genes have also been identified in *M. truncatula* and Arabidopsis that regulate longevity during seed maturation (Righetti et al., 2015). For instance, TFs such as WRKY3 and NFLX1 involved in defense responses of plants were also known to affect seed longevity by modulating seed coat permeability (Debeaujon et al., 2000). Similarly, the lignin content of soybean testa has been linked to seed permeability and mechanical damage resistance (Capeleti et al., 2005). Although, seed mucilage is expected to play a variety of roles in seed physiology, its role in seed longevity remains unclear. Since the presence of a large gelatinous mass of mucilage in the seed could act as an additional blockade to the cell layers, its role in affecting seed longevity needs to be investigated in the future.

## Candidate Genes/Proteins Affecting Seed Longevity in Legumes

The delineating physiological and genetic factors influencing the seed longevity have been very well-elucidated in model crop species *Arabidopsis thaliana*. Whereas in case of legumes, studies were concentrated mainly on *Medicago truncatula* (Rose and Rose, 2008), because of the availability of complete genome information, spatio-temporal expression data, collection of large germplasm, and different types of mutants (Young et al., 2005; Bandyopadhyay et al., 2019). Among the other common legumes, study of seed longevity has been deciphered mainly in pea, soybean, common bean, *Vicia* sp. and chickpea. Inferring the synteny between model crop species with other leguminous crops, based on the genes involved, as well as genomic and post-genomic approaches could facilitate identification of novel candidate genes and proteins that play a vital role in modulating seed longevity.

### Genes Involved in Protection of Seed Longevity

Several genes and gene products were known to be associated in enhancing desiccation tolerance as well as preservation of seed longevity during storage as well as late seed maturation phase. At the late seed maturation stage of seed development, seeds acquire two key adaptive traits that include dormancy and longevity. Both the traits are critical and play complementary roles in maintaining the life of embryo, till seed attains favorable conditions and ensure germination. In case of legumes, longevity is progressively acquired during seed maturation from seed filling onwards (Lima et al., 2017). The increase in longevity has been associated with the accumulation and expression of HSFs, LEA-proteins, soluble non-reducing sugars of raffinose family (Gaff et al., 2013; Leprince et al., 2017; Lima et al., 2017; Marques et al., 2018). Apart from this, chlorophyll degradation in seeds is considered as vital step that take place during seed maturation and storage, since residual chlorophyll compromise the quality of seeds in terms of longevity. All the above-mentioned processes, alone or in combination regulate a cascade of events by triggering and down-regulating the activities of various genes and TFs that could affect seed specific processes controlling longevity (Table 1).

**Table 1:** Candidate genes/proteins responsible for imparting desiccation tolerance and longevity in seeds during maturation and storage.

**Candidate genes/protein**	**Crop species**	**Function**	**References**
ABI3	*M. sativa*	Involved in synthesis of LEA proteins and impart desiccation tolerance	Delahaie et al., 2013
MAT9	*G. max*	Impart cryoprotective ability to seeds; but role in seed longevity is unknown	Momma et al., 1997, 2003
HSF9A	*M. truncatula*	Controls the process of seed aging when the seeds are exposed to sub-optimal storage conditions	Zinsmeister et al., 2020
		Deregulation of genes involved in ABA catabolism, biosynthesis and signaling; negative regulator for determining depth of dormancy	
GolS1_A and GolS2_B	*G. max*	Knock-down expression of the genes could reduce the synthesis of RFO in seeds	Le et al., 2020
RS1, RS2 and RS3	*G. max*	Involved in synthesis of raffinose	Dierking and Bilyeu, 2008
SS	*G. max*	Involved in synthesis of stachyose	Qiu et al., 2015
RS2	*G. max*	Knock-down expression of the gene induced the reduction of raffinose and stachyose	Valentine et al., 2017
RS2	*P. vulgaris*	Key candidate gene involved in the synthesis of RFOs in seeds	de Koning et al., 2021
GolS1_A, RS2_A and RS2_B	*G. max*	Potential candidate genes for RFOs synthesis in seeds	
GolS	*C. arietinum, V. hirsute* and *P. sativum*	Involved in maintenance of seed vigor and longevity and acquisition of desiccation tolerance	Peterbauer et al., 2001; Gojło et al., 2015; Salvi et al., 2016
SNF4b	*M. sativa*	Regulator of RFOs synthesis and mutants of the gene displayed reduction in seed longevity	Rosnoblet et al., 2007
DOG1	Many crop species	Positive regulator involved in the synthesis of RFOs, LEA, and HSPs promoting seed longevity and dormancy	Dekkers et al., 2016; Chahtane et al., 2017; Nonogaki, 2019
ABI5	*M. sativa* and *P. sativum*	Controls gene modules associated to RFO and LEA synthesis and chlorophyll degradation	Dekkers et al., 2016; Zinsmeister et al., 2016
GSH	*P. sativum*	Redox status of the gene plays a vital role in seed aging process	Chen et al., 2013
FPG/OGG1	*M. sativa*	Expression of the genes could repair the DNA damage acquired during seed storage	Macovei et al., 2011
LIG1	*Medicago* and Arabidopsis species	Associated with double strand break repair and maintenance of seed longevity	Righetti et al., 2015
PIMT	*C. arietinum*	Involved in the repair of protein damage	Verma et al., 2013
MSR	*M. truncatula*	Involved in repair of oxidized proteins and reduction of methionine sulfoxide residues	Chatelain et al., 2013

#### Accumulation of LEA Proteins

At the late seed maturation phase, dehydration of seed cells occurs and activates a set of genes encoding hydrophilic proteins, which are popularly known as LEA proteins. Since, they accumulate in seeds during the late maturation stage, they are detected only in dry seeds and are not available after germination (Amara et al., 2014). They could stabilize the glassy cytoplasm in combination with sugars and play an important role in maintaining seed viability (Amara et al., 2014; Artur et al., 2019). LEA proteins are divided into eight distinct sub-classes on the basis of their amino acid sequences, phylogenetic relationships and repeated motifs as LEA1, LEA2, LEA3, LEA4, LEA5, LEA6, dehydrin (DHN), and seed maturation protein (SMP) (Battaglia and Covarrubias, 2013).

Several *in vivo* studies documented that expression of LEA genes in the vegetative tissues of wide range of species, offers enhanced tolerance against different stresses (Figueras et al., 2004; Bahieldin et al., 2005; Xiao et al., 2007; Olvera-Carrillo et al., 2010; Roychoudhury and Banerjee, 2015). In Arabidopsis, knock-down expression of the gene of the LEA1 group led to decrease in drought tolerance in the seedlings and reduction in the ability to recover after stress (Olvera-Carrillo et al., 2010). It is noteworthy to mention that, the degree of environmental stresses to which plants are exposed are often much milder than those experienced by developing seeds during maturation drying. Thus, the role of LEA proteins in seed development for imparting desiccation tolerance and longevity remains largely elusive. A study on *Medicago truncatula* revealed that expression of few, but not all LEA proteins is essential for achieving desiccation tolerance (Boudet et al., 2006). Comparison of LEA proteome of orthodox (*M. truncatula*) and recalcitrant (*Castanospermum australe*) legume species revealed 16 homologous genes out of the 17 genes detected, belonging to LEA1, LEA4, LEA5, DHN, and SMP groups. Among them, polypeptides of 12 genes were either reduced or strongly reduced in the recalcitrant species as compared to the orthodox species. Further, the role of gene ABI3 in synthesis of LEA proteins has been assessed in *M. sativa*. Characterization of LEA proteome in the mutants of *Mt*ABI3 revealed severe reduction in the accumulation of LEA transcripts imparting desiccation sensitivity to the orthodox seeds that explains, LEA genes are highly ABI3 responsive (Delahaie et al., 2013). Manfre et al. (2006, 2009) identified that mutation of the Arabidopsis gene early methionine 6 (*At*EM6) (class 1 LEA protein) in seeds could alter the timing for initiation of desiccation tolerance. However, the protein was not reported to be essential for imparting desiccation tolerance in the seeds as the mutants produced viable dry seeds (Manfre et al., 2009).

Another group of LEA proteins, group II or DHNs are presumed to protect cellular macromolecules against injuries caused by stressors (Hara, 2010). They are considered as essential phytomolecules that accumulate mostly during the late phases of seed development, and can also be synthesized in the vegetative tissues in response to extreme stress factors (Hong-Bo et al., 2005). They are considered to play a critical role in the conservation of seed longevity (Delahaie et al., 2013). Since they are believed to be synthesized during late maturation phase of seed development, they are conventionally present in orthodox seeds. Extensive research evidences suggest that DHNs are key players for maintenance of viability in orthodox seeds (Kleinwachter et al., 2014; Azarkovich, 2020; Solberg et al., 2020). Hundertmark et al. (2011) developed an RNA-interference (RNAi) construct against the seed-expressed dehydrin gene LEA14 of Arabidopsis that was introduced into wild-type plants. This affected the transcripts of two other dehydrin homologs *viz*., XERO1 and responsive to abscisic acid 18 (RAB18) along with the target gene. However, knock-down expression of these genes revealed no significant effect on desiccation tolerance and the mature dry seeds upon rehydration germinated normally. But reduction in the activity of these dehydrin genes exhibited 2-fold decline in the longevity. In soybean, another group 2 LEA gene, maturation-associated protein 9 gene (MAT9) was obtained from mature soybean seeds that could produce acid soluble 26-kDa (AS26k) polypeptide imparting cryoprotective stability to the seeds, however its pertinent role in desiccation tolerance and longevity was not discussed (Momma et al., 1997, 2003).

In contrast, recalcitrant seeds bypass the maturation phase and cannot synthesize DHNs. As an exception, certain atypical recalcitrant species e.g., *Quercus robur* and *Euterpe edulis*, were reported to undergo maturation drying phase alike orthodox seeds and the corresponding water loss could induce the synthesis of DHNs (Finch-Savage et al., 1994; Gee et al., 1994; Greggains et al., 2000). In analogy to this, accumulation of two dehydrins BudCar5 and DHN-cognate was reported to be higher in desiccation sensitive species (*C. australe*) as compared to its expression in desiccation tolerant species (*M. truncatula)*. Proteomic analysis revealed that DHNs comprise 83% of the LEA proteome in *C. australe*, while in *M. truncatula*, their expression was restricted to only 20% (Delahaie et al., 2013). Thus, further investigation is essential to speculate the functional role of dehydrins in desiccation sensitive or recalcitrant species.

#### Activity of HSFs

In legumes, alike LEA proteins, HSPs and small HSP (sHSP) accumulate during the acquisition of dormancy and longevity, at the late seed maturation phase and retain in their native state in dry seeds (Kalemba and Pukacka, 2007; Sano et al., 2016; Leprince et al., 2017). The ability of sHSP to form large oligomeric complexes, enable them with high chaperone activity (Kalemba and Pukacka, 2007). Thus, sHSP could enhance the folding of newly synthesized proteins as well as refolding of polypeptides with damaged tertiary structure and also assist in fighting against ROS in seeds (Kalemba and Pukacka, 2007; Kaur et al., 2015; Sano et al., 2016; Leprince et al., 2017).

Accumulation of transcripts of various sHSPs during seed maturation as well as in dry seeds were reported in several plant species including pea (DeRocher and Vierling, 1994). The transcript levels of HSPs *viz*., HSP17.4 and *Os*HSP18.2 were reported to be upregulated in Arabidopsis and rice respectively, during the seed maturation stage enhancing the acquisition of dormancy and longevity (Wehmeyer and Vierling, 2000; Kumar et al., 2009; Kaur et al., 2015). Among the various HSFs, HSFA9 is a unique member of the HSFA family and specifically expressed in seeds during development. HSFA9 remains under the regulatory control ABI3 and is activated without the need of a heat shock (Kotak et al., 2007). Overexpression of sunflower HSFA9 in tobacco activated the expression of several other HSFs. The transgenic seeds expressed increased resistance against seed deterioration, deciphering the specific role of this HSFs in enhancing seed longevity (Tejedor-Cano et al., 2010). Verdier et al. (2013) identified a homolog of HSFA9 to be involved in regulating longevity and desiccation tolerance in *M. truncatula*. In another study, Zinsmeister et al. (2020) reported that HSFA9 does not play an important role in seed longevity, especially when the seeds are exposed to optimal storage conditions. Whereas, subjecting the seeds to high humidity could promote aging and loss of viability in HSFA9 mutants of *M. truncatula*. The study concluded that HSFA9 mutants have comparable lifespan alike wild types under mild storage conditions. Nevertheless, HSFA9 could control the process of seed aging and exert effects on seed longevity when the seeds are exposed to hot and humid storage conditions. During wet and hot conditions of storage, the expression of sHSP having chaperone activity was reported to be enhanced by HSFA9. Along with sHSP, deregulation of other genes *viz*., HSP70, HSFB2A, and HSFA2, as well as rotamase FKBP 1 (ROF1) and bcl-2-associated athanogene 6 (BAG6) has been noticed (Zinsmeister et al., 2020).

#### Synthesis of RFO's

Switching off cellular metabolism and respiratory processes is critical for survival of seeds in the dry state particularly during storage. Few research studies strongly suggest higher levels of metabolism as a characteristic feature linked to desiccation sensitivity, while controlled or decreased metabolic functions to be associated with desiccation tolerance (Kijak and Ratajczak, 2020). It has been widely reported that the physio-chemical properties of RFOs could impart desiccation tolerance to orthodox seeds. These sugars mainly comprise of sucrose and raffinose family of oligosaccharides, popularly known as RFOs. Initially, during the process of dehydration, oligosaccharides along with LEA proteins substitute the water present in the polar heads of phospholipids with hydrogen bonds (Hoekstra et al., 2001). This restructures the liquid state of membrane into gel phase. The loss of water molecules decrease the mobility of lipids accompanied by stronger van der Waals interactions between lipid heads (Hoekstra et al., 2001; Buitink and Leprince, 2008). Subsequent to this, the cytoplasm of dehydrated cells becomes glassy instead of converting into solid state. Thus, the viscosity of cytoplasm enhances and diffusion of water and oxygen gets suppressed, resulting in the overall reduction of metabolism in the cells of seeds (Hoekstra et al., 2001; Buitink and Leprince, 2008; Walters, 2015). Along with this, the activities of several oligosaccharides are effective against cell membrane damage and oxidative damage imposed in seed cells during dry storage. The glassy state promoted by the RFOs is capable of retarding the Maillard reactions that are involved in lipid peroxidation and decline of seed vigor.

In leguminous crops, RFOs are mainly stored in the seeds under normal growth conditions. They are synthesized *de novo* during the seed maturation and protect the seed against desiccation, enhancing seed longevity during storage (Blochl et al., 2007; Gangola et al., 2016). Buitink et al. (2000) reported no direct causal relationship between cytoplasmic glassy state induced by RFOs with the seed longevity. But, other alternative role of RFOs in establishment of seed longevity could be assigned to the function of carbon storage. This function of RFOs facilitate mobilization of energy during germination after aging, and assist in mitigating environmental stress as well as provide protection against oxidative damage (Nishizawa et al., 2008). Few studies hypothesized that decline in the synthesis of RFOs may not have a significant impact on seed vigor or large-scale synthesis of RFOs is not really essential for seed longevity (Bentsink et al., 2000; Neus et al., 2005).

In the recent past, many research studies revealed prominent role of RFOs in controlling seed traits, especially desiccation tolerance and longevity. Buitink and Leprince (2008) reported decline in the process of seed aging with increased cellular viscosity and restricted molecular mobility, over a broad range of moisture and temperature regimes. This indicates that seed processes associated to deterioration remain under the control of molecular mobility. In plants, four different members of the RFO family were reported *viz*., raffinose, stachyose, verbascose, and ajugose (Peterbauer et al., 2001). Soybean and common bean mainly synthesize raffinose and stachyose, and verbascose to a minimal extent in their seeds (Yamaguishi et al., 2009; Valentine et al., 2017). The synthesis of RFOs depends on the influx of galactinol that acts as galactosyl donor. Along with galactinol, stachyose and galactosyl cyclitols could also be used as galactosyl donors (de Koning et al., 2021). The synthesis of galactinol, stachyose and galactosyl cyclitols has been identified to be catalyzed by the enzymatic activities of galactinol synthase (GolS) and stachyose synthase (SS) genes respectively. During the initiation of RFO synthesis, GolS catalyses the transfer of galactose from UDP-galactose to myoinositol forming galactinol. Next to this, synthesis of raffinose takes place, by transfer of galactosyl moiety to sucrose from galactinol that is mediated by raffinose synthase (RS). This reaction is reversible in nature and produces the first RFO during the RFO biosynthesis pathway (Peterbauer et al., 2001). Subsequent to this, RFOs with high degree of polymerization (DP) that possess more than three monosaccharides are synthesized by further addition of galactose moieties, donated by galactinol, to lower DP RFOs. For instance, both stachyose and verbascose are produced from the enzymatic activity of SS (Peterbauer et al., 2002).

Le et al. (2020) identified six GolS genes in soybean *viz*., *Gm*GolS1_A, *Gm*GolS1_B, *Gm*GolS2_A, *Gm*GolS2_B, *Gm*GolS3_A, and *Gm*GolS3_B. The study found that knock-down expression of two GolS genes (*Gm*GolS1_A and *Gm*GolS2_B) could significantly reduce the total RFO content by 35.2% in soybean seeds affecting longevity. Dierking and Bilyeu (2008) reported three RF genes involved in synthesis of raffinose *viz*., *Gm*RS1, *Gm*RS2, and *Gm*RS3 in soybean. Qiu et al. (2015) identified one gene *Gm*SS to be responsible for stachyose synthesis. Valentine et al. (2017) reported reduction in the content of raffinose (0.63%) as well as stachyose (3.79%) in soybean seeds of wild type to 0.11 and 1.21% respectively, using a silenced gene construct targeting an isoform of single RS gene i.e., *Gm*RS2. In analogy to the above reported studies, de Koning et al. (2021) also reported six genes of GolS, three genes of RF and one gene of SS to be involved in RFOs production of soybean. In addition to this, the study reported three GolS genes (*Pv*GolS1, *Pv*GolS2, and *Pv*GolS3), two RS genes (*Pv*RS1 and *Pv*RS2) and one SS gene (*Pv*SS) to be involved in the synthesis of RFOs in case of *Phaseolus vulgaris*. The study cited tissue-specific gene expression patterns in both soybean and common bean. In case of soybean, genes *Gm*GolS1_A, *Gm*RS2_A, and *Gm*RS2_B were identified as potential targets for modulating RFO synthesis in seeds, and in common bean *Pv*RS2 was found to be the candidate gene involved in affecting seed longevity. de Souza Vidigal et al. (2016) reported that galactinol can serve as a biomarker for assessment of longevity in mature dry seeds, particularly in tomato and the members of Brassicaceae.

**Figure 3:**
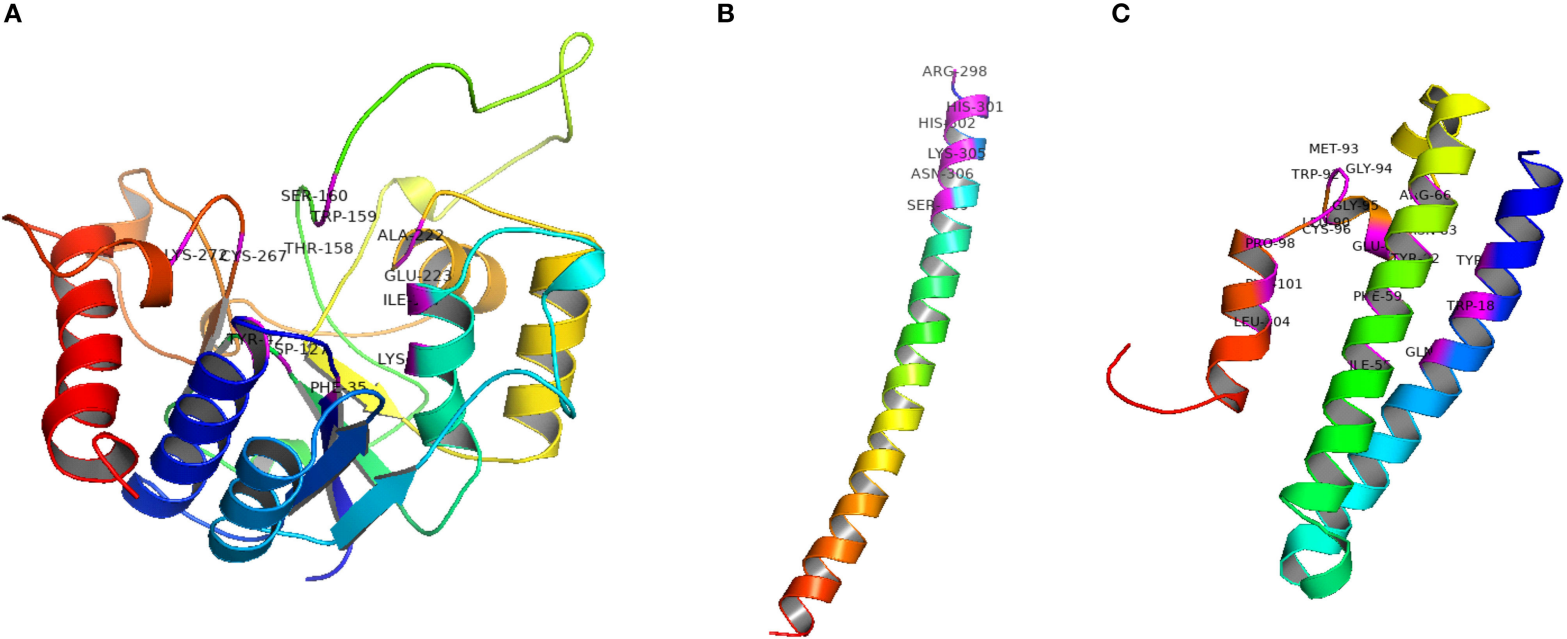
3D structure of candidate genes/proteins associated with seed longevity in legumes. **(A)** GolS1 (Active site residues are Ile107, Lys111, Asp127, Thr158, Trp159, Ser160, Ala222, Glu223, Cys267, Lys272, Phe35, Ala37, Tyr42 **(B)** ABI5 (Active site residues are Arg298, His301, His302, Lys305, Asn306, Ser309), **(C)** DOG1 (Active site residues are Phe101, Leu104, Tyr15, Trp18, Met19, Gln22, Ile55, Phe59, Tyr62, Arg66, Tyr77, Asn83, Glu87, Leu90, Trp92, Met93, Gly94, Gly95, Cys96, Pro98).

Salvi et al. (2016) reported GolS to play an important role in seed vigor and longevity of chickpea. Similar observations were also reported in *Glycine max* and *Vicia hirsuta*, suggesting their participation in seed desiccation tolerance and longevity (Castillo et al., 1990; Obendorf et al., 2009; Gojło et al., 2015). The galactinol synthase enzymes of chickpea were identified to be encoded by two genes (*Ca*GolS1 and *Ca*GolS2), of which *Ca*GolS1 revealed predominant expression in developing and mature seeds. Similar pattern of tissue-specific expression was also observed in few other species including *Pisum sativum*, soybean and *Vicia hirsuta*, where GolS1 gene was observed to be induced during seed development, enhancing seed desiccation tolerance (Peterbauer et al., 2001; Gojło et al., 2015; de Koning et al., 2021). Transgenic expression of the *Ca*GolS1 gene triggered redox homeostasis through ROS scavenging, and restriction of both lipid peroxidation and cellular damage. This in turn, promoted the desiccation tolerance, vigor and longevity of chickpea seeds. The 3D structure of the GOS1 protein is projected with the help of Swiss model and presented in Figure 3A. The active sites of GolS1 protein were predicted to be made of Ile107, Lys111, Asp127, Thr158, Trp159, Ser160, Ala222, Glu223, Cys267, Lys272, Phe35, Ala37, and Tyr42 residues. Apart from these studies, Rosnoblet et al. (2007) provided direct evidence of link between RFOs metabolism and longevity, mediated by ubiquitous sucrose non-fermenting-like kinase (*Sn*RK1) gene. It is well-established that, *Sn*RK1 coordinates and adjusts metabolic demands during process of seed maturation (Radchuk et al., 2010). In *Medicago* species the mutants of *Mt*SNF4b, a regulatory subunit of *Sn*RK1 complex, failed to synthesize RFOs that resulted in decline of seed longevity. This suggests that *Sn*RK1 complexes in seeds are responsible for improving seed longevity by affecting the non-reducing sugar metabolism during the later stages of seed maturation (Rosnoblet et al., 2007).

Recent research evidences suggest that, along with accumulation of RFOs in the seeds, distribution of accumulated RFOs among different compartments of the cell have been proven to have a considerable impact on enhancing seed longevity. Hell et al. (2019) reported differences in the concentration of different sugars among subcellular compartments. Wherein, increase of sugars has been noticed in the cytosol of mature cotyledons, as compared to stages I and VI of seed development in *Erythrina speciosa*. But in case of Arabidopsis, Knaupp et al. (2011) found a protective function of RFOs in plastids, but not in the cytosol. Thus, allocation of sucrose and RFOs during seed maturation phase is also defined to play a prominent role in determining the seed longevity.

#### Effect of Chlorophyll Genes in Acquisition of Longevity

Chlorophyll retention in the seeds of many crop species appears to be detrimental for seed longevity. Generally, chlorophyll is degraded during the late stages of seed maturation, before seed reaches the dry state (Nakajima et al., 2012; Delmas et al., 2013; Teixeira et al., 2016). Arabidopsis green-seeded (GRS) mutants that retained twice the chlorophyll content as the wild types, exhibited significant reduction in storability (Clerkx et al., 2003). Similar to this, characterization of double mutants of non-yellow coloring1 (NYC1) and nyc1-like (NOL), demonstrated significant defects in chlorophyll degradation. The mutant seeds contained 10-fold more chlorophyll than the wild type Arabidopsis and displayed negative impact on longevity (Nakajima et al., 2012). In seeds of Arabidopsis, degradation of chlorophyll has been identified to be controlled by the activity of ABI3, NAC transcription factor (NAC016), and ABA-responsive element binding factor (ABF4); (Nakajima et al., 2012; Delmas et al., 2013). Dekkers et al. (2016) analyzed the double mutants of DOG1-1, ABI3-1 and revealed that, DOG1 with the interaction of ABI3 could control the accumulation of seed storage proteins and chlorophyll degradation in Arabidopsis. Another evidence of effect of chlorophyll retention on seed longevity was provided by Zinsmeister et al. (2016) in pea and *M. truncatula*. The ABI5 mutant seeds of both the species reported retention of chlorophyll and decline in longevity, whereas the effects were more pronounced in pea mutants.

Investigation on the cause-effect relationship between seed longevity and chlorophyll content revealed two plausible reasons affecting the processes. The first explanation assumes that, impaired chlorophyll degradation could lead to accumulation of phototoxic products and chlorophyll catabolites, resulting in photo-oxidative damage in seeds. However, Canfield et al. (1995) reported very minimal or traces of chlorophyll catabolites in the seeds of the stay-green mutants of soybean *viz*., green cotyledon (cytG) and Gd1d2 that retain chlorophyll. This suggests that accumulation of phototoxic intermediates to such minimal extent may not impair the longevity. The second hypothesis that correlates chlorophyll retention and longevity suggests that, incomplete regulation of the chloroplast dedifferentiation could lead to oxidative damage compromising seed viability. In accordance with this hypothesis, Zinsmeister et al. (2016) identified that the ABI5 could indirectly repress the photosynthesis-associated nuclear genes (PhANGs) instigating progressive shutdown of photosynthesis in seeds, prior to reaching the quiescent dry state. Thus, ABI5 in legumes has been speculated to enhance chlorophyll degradation and impair photosynthesis during seed maturation to avoid oxidative stress and promote seed longevity.

#### Maintenance of ROS Homeostasis During Storage

During storage, seeds are often exposed to high RH and high temperature that results in rapid decline of seed longevity. This could be possibly due to oxidation of macromolecules induced by the activity of ROS. ROS are anticipated to play a dual role in seed physiology. At substantial rates, they play the key role of signaling molecules in enhancing seed germination; whereas on the other hand, excess ROS impose severe oxidative stress. Due to their high reactivity, they are capable of inducing chain reactions and cause irreversible oxidative damage to most proteins, lipids and DNA (Harman and Mattick, 1976; Bailly, 2004). In such cases, longevity is conferred by antioxidant systems that play an important role in redox regulation by removal of excess ROS (Lima et al., 2017). The antioxidant systems include both non-enzymatic ROS scavenging systems and enzymatic ROS detoxification systems. The enzymatic antioxidants such as superoxide dismutase (SOD), catalase (CAT), ascorbate peroxidase (APX), and monodehydroascorbate (MDAR) and glutathione reductase (GR) were deciphered to play a key role in the removal of ROS accumulated during storage, as well as control free radical overproduction upon imbibition (Bailly, 2004; Kumar et al., 2015). Whereas, the non-enzymatic ROS scavenging systems include seed storage proteins and low molecular weight antioxidants such as tocopherols, ascorbate and glutathione that play an important role during seed development and maturation. Although, many studies were conducted to identify the role of antioxidant genes controlling seed longevity in Arabidopsis and other crop species, very few studies were attempted in legumes. Since, legumes possess homologs of most of the genes identified in Arabidopsis, they are presumed to perform similar action in controlling seed longevity of legumes.

Seed aging is correlated with a decline in the cellular antioxidant potential followed by a corresponding increase in the concentration of ROS. Renard et al. (2020) identified few key genes involved in ROS generation and scavenging that could affect seed longevity in Arabidopsis mutants. Among them, respiratory burst oxidase homolog (RBOH) produced ROS was identified as the major source of deterioration during aging, and downregulation of RBOH gene could enhance seed longevity. Further, knock-down expression of the genes dehydro-ascorbate reductase 1 (DHAR1) and photosystem I subunit D1 (PSAD1) reduced the seed longevity. These two genes were anticipated to play a key role in ROS detoxification process. DHARs are involved in catalyzing the regeneration of ascorbate oxidized during H_2_O_2_ scavenging (Foyer and Noctor, 2011) and PSAD is involved in balancing photosystems, that otherwise leads to excessive ROS synthesis (Pinnola and Bassi, 2018). Another candidate gene NADP-dependent malic enzyme (NADP-ME) was identified in barley, that is involved in protecting the seeds from oxidation during storage and the mutants lacking functional malate dehydrogenase displayed reduced viability (Yazdanpanah et al., 2019; Shvachko and Khlestkina, 2020). The role of NADP-ME in the biosynthesis of flavonoids and lignin as well as ROS metabolizing enzymes has already been discussed in many studies (Corpas and Barroso, 2014; Heyno et al., 2014; Chen et al., 2019).

Among the various non-enzymatic antioxidants, reduced glutathione (GSH) plays a major role in the regulation of the intracellular redox environment, and is being used as a tool for estimation of seed viability (Kranner et al., 2006). In accordance to this, Nagel et al. (2015) suggested that, GSH could serve as a robust marker of seed deterioration in barley. Chen et al. (2013) reported that glutathione redox state could play an important role in aging of pea seeds. Another vital non-enzymatic antioxidant that plays an important role in maintaining seed viability is tocopherol (vitamin E). It was identified to be involved in preventing non-enzymatic lipid oxidation during seed storage. In Arabidopsis, mutants of the gene vitamin E deficient 1 (VTE1) reported defects in the biosynthesis of tocopherols, thereby increasing lipid peroxidation and reducing seed longevity (Sattler et al., 2004). However, Giurizatto et al. (2012) identified a linear correlation of α-tocopherol with storage time, and artificial aged seeds had higher levels of α-tocopherol than the naturally aged seeds of soybean. Along with tocopherols, lipocalins have been reported to prevent lipid peroxidation and they both act in a synergistic manner to prevent seed aging (Havaux et al., 2005; Boca et al., 2014).

Seed storage proteins (SSPs) have been described as indicators of seed aging (Job et al., 2005; Arc et al., 2011). 12S storage protein subunits are the major storage protein of dicot seeds (Rajjou et al., 2008). They serve as a primary target for oxidation in seeds, and 12S globulin α-subunits of SSPs are preferentially involved in carbonylation (irreversible oxidation) process. Mutants that possess defects in 12S globulin genes displayed reduction in seed longevity in Arabidopsis (Nguyen et al., 2015). The role of SSPs in seed longevity was also corroborated by the finding of 12S (Cruciferins) protein sub unit, the most abundant SSPs in Arabidopsis. The seeds of a triple mutant for three CRU isoforms (crua, crub, and cruc) were more sensitive to artificial aging and their seed proteins were highly oxidized compared to wild-type seeds (Nguyen et al., 2015). Along with this, SSPs were proposed to maintain ROS homeostasis protecting cellular components. The ROS scavenging activity of SSPs has been found to be associated to a chaperone function (Nguyen et al., 2015). Since, the relationship between ROS scavenging and seed aging has been well-established in different crop species, further investigation and identification of homologs for these genes in leguminous species could make them reliable markers for seed deterioration. Considering the abundance and higher affinity of SSPs toward oxidation, it is hypothesized that they could serve as one of the effective apparatus for scavenging excess ROS and protects cellular structures as well as other seed proteins (Sano et al., 2016).

### Genes Involved in the Repair/Reversal of Seed Deterioration During Storage

Aging processes provoke changes in the structural and functional properties of membrane lipids, key proteins and nucleic acids. Thus, reversal of damage or repair is critical for maintenance of seed germination and longevity (Balestrazzi et al., 2011; Waterworth et al., 2015; Kurek et al., 2019). The ability of aged seeds to germinate provides direct evidence that DNA repair mechanisms are activated in the embryo during early germination (Balestrazzi et al., 2011). During seed storage, stochastic lipid oxidation produces several peroxides, hydroperoxides, carbonyl, and nitrosyl groups. These compounds upon interaction with molecules in the close proximity enhance further oxidation, fragmentation and formation of adducts. In legume seeds, the downstream products of lipid peroxidation such as propanal, butanal, and hexanal were reported to be progressively accumulated during storage (Mira et al., 2010; Colville et al., 2012). In pea seeds, the increase in propanal and hexanal concentrations was found to be associated with loss of viability (Colville et al., 2012). In contrast to this, in *Lathyrus pratensis* no apparent correlation between rate of aging and apparent increase in by-products of lipid peroxidation was reported. It was hypothesized that seed aging may not be affected through lipid peroxidation by-products, suggesting these compounds are not the actual cause of deterioration rather they are the consequence of the peroxidation process (Mira et al., 2016). However, few authors documented complex changes in the lipidome and oxylipidome profiles of seed after storage, involving lipids both from the membranes and oil reserves (Nagel and Borner, 2010; Boca et al., 2014; Wiebach et al., 2020). Hence, further work is needed to understand factors and genes triggering the pathways leading to lipid oxidation and hydrolysis, and how it could affect the seed germination after aging.

Seed aging is often associated with progressive accumulation of DNA damage in the embryo including modifications or loss of nucleotides, SSBs and DSBs (Cheah and Osborne, 1978; Dourado and Roberts, 1984; Cordoba-Canero et al., 2014). Thus, cellular survival depends on the concerted action of powerful repair pathways for reverting the base damage and DNA strand breaks. During accelerated aging, the most prevalent form of base damage that occurs, is the oxidation of 8-oxoguanine (8-oxoG) (Chen et al., 2012). Removal of 8-oxoG is mediated by the activity of either formamidopyrimidine-DNA glycosylase (FPG), or 8-oxoguanine DNA glycosylase/lyase (OGG1) (Cordoba-Canero et al., 2014). Expression of both the genes has been reported to be increased in *M. truncatula* seeds during imbibition (Macovei et al., 2011). Arabidopsis seeds over-expressing OGG1 reported significant reduction in DNA damage imposed due to the formation of 8-oxoG (Chen et al., 2012). If severe damage to DNA occurs with steric changes in the DNA duplex structure, NER mechanism will become operational, wherein oligonucleotides of ~30 bases will be excised and DNA polymerase fills the gaps in the single stranded region. In *Phaseolus vulgaris*, expression of genes involved in NER was found to be increased toward the end of seed development, that may prime the seeds with repair factors required during early imbibition and promote viability (Parreira et al., 2018). In Medicago and Arabidopsis, DNA ligase 1 (LIG1) gene linked with DSB repair was reported to be associated with maintenance of seed longevity (Righetti et al., 2015). Analysis of DNA ligase mutants with a loss of DNA repair function established the genetic link between DNA repair and seed longevity (Charbonnel et al., 2011). Along with this, few key genes were reported to play pivotal role in controlling cell cycle progression in the presence of DNA damage (Sancar et al., 2004). These genes were thought to be involved in activation of cell cycle checkpoints, DNA repair factors, programmed cell death (PCD) and endoreduplication (Fulcher and Sablowski, 2009; Adachi et al., 2011). Among them, the protein kinases ataxia telangiectasia mutated (ATM) and ATM and rad3-related (ATR) function as master regulators of the cellular response to DNA damage in seeds (Waterworth et al., 2016). Further, PCD response in the damaged tissues was also found to be associated with the activity of ATM and ATR kinases (Fulcher and Sablowski, 2009; Furukawa et al., 2010). Both kinases act through a transcription factor suppressor of gamma response1 (SOG1) that is proposed to have key role in the resumption of embryo growth during germination followed by genome damage (Johnson et al., 2018). In general, the seed cells remain in G1 phase prior to germination and the contribution of PCD to seed vigor remains unclear. The appearance of DNA laddering, a characteristic hallmark of PCD has been detected in both sunflower and pea seeds subsequent to aging and seed deterioration (El-Maarouf-Bouteau et al., 2011; Chen et al., 2013). The marks of cell death observed during seed deterioration drives the possible role of PCD in maintenance of viability, and PCD is anticipated to take place in cases where damage to cellular components exceeds repair capacity (Kranner et al., 2010).

In comparison to DNA, RNA is more vulnerable to ROS oxidation owing to its structural features. Since, dry seeds store large amount of mRNAs, damage to mRNA blocks the translation in imbibed seeds that affects seed longevity (Rajjou and Debeaujon, 2008). In aged seeds of *Pisum sativum*, loss of germination ability was accompanied by a reduction in the concentration and integrity of RNA (Kranner et al., 2010). Fleming et al. (2017) assessed the fate of RNA in dry seeds of soybean and various degrees of transcript fragmentation were reported in the seeds. Nevertheless, few transcripts of mRNA remained intact during seed aging that were related to ribosomal functions (Rajjou and Debeaujon, 2008; Galland et al., 2014). Similar to this, knockout lines for the elongation factor eIF(iso)4G1 and eIF(iso)4G2 synthesized from the stored seeds of Arabidopsis showed a severe reduction in longevity (Lellis et al., 2010). These findings emphasize that various components of transcription, especially elongation factors contribute to the seed survival in the dry state. So far, the mechanisms regulating transcript fragmentation and pathways that lead to loss of seed germination remain unknown. The process of RNA oxidation was anticipated to be strongly involved in the process, wherein differential sensitivity to aging has been reported, according to the RNA species. In sunflower, increase of oxidized mRNA in the form of 8-hydroxyguanosine (8'OHG) had an impact on releasing dormancy (Bazin et al., 2011). In contrast, in soybean no apparent correlation between the amount of 8'OHG mRNA and loss of viability has been noticed (Fleming et al., 2017).

Similar to DNA and RNA, proteins also tend to undergo oxidation or covalent modifications which often leads to the loss of function (Rajjou and Debeaujon, 2008). Seed longevity has also been shown to be associated with protein repair systems mediated by the activity of PIMT (Oge et al., 2008) and methionine sulfoxide reductases (MSR) (Chatelain et al., 2013). PIMT is involved in reversal of spontaneous covalent modification by converting L-aspartyl or asparaginyl residues to abnormal isoaspartyl (isoAsp) residues, whereas MSR repairs the oxidized proteins and reduces methionine sulfoxide residues (Weissbach et al., 2005). Overexpression of chickpea PIMT genes, *Ca*PIMT1 and *Ca*PIMT2 in the seeds of Arabidopsis demonstrated enhanced seed longevity suggesting PIMT-mediated protein repair is critical for seed longevity (Verma et al., 2013). Similar to this, the activity of MSR has been positively correlated with the longevity of seed lots in *M. truncatula* genotypes (Chatelain et al., 2013).

### Phytohormone-Mediated Regulation of Seed Longevity

Phytohormones are involved in the regulation of seed maturation and underlying developmental pathways, and thereby control most of the seed quality parameters including seed germination, dormancy and longevity. Among plant growth regulators, ABA plays an important role in developmental regulation of seed longevity, dormancy and desiccation tolerance (Clerkx et al., 2004). During seed maturation, a network of transcription factors known as LAFL network comprising of leafy cotyledons 1 (LEC1), ABA insensitive 3 (ABI3), fusca 3 (FUS3), and leafy cotyledons 2 (LEC2) (Lepiniec et al., 2018) act together to regulate the maturation process. These TFs were identified to control the expression of genes involved in seed maturation and onset of desiccation tolerance. For instance, mutants of ABI3, LEC and FUS3 exhibited reduced seed storage, desiccation intolerance and precocious germination resulting from incomplete seed maturation and early transition to vegetative stage (North et al., 2010). Similar to this, double mutants of ABI3 and LEC1 lost their viability within one week after harvest (Sugliani et al., 2009). When the mutants of ABI3 and LEC1 were replaced with wild type allele through backcrossing for six generations, they could restore the seed longevity (Sugliani et al., 2009). These results emphasize that ABI3 and LEC1 could regulate directly or indirectly several sHSPs, LEA proteins and some SSPs that are associated with longevity (Hundertmark et al., 2011; Chatelain et al., 2012). Among the various transcription factors, ABI3 is believed to play a key role in triggering the expression of HSFA9 by activating its promoters and confer desiccation tolerance (Kotak et al., 2007; Verdier et al., 2013). Along with ABI3, another basic leucine zipper (bZIP) TF ABI5 has been considered as a principal regulator of late seed maturation in legumes (Dekkers et al., 2016; Lepiniec et al., 2018). The seeds of ABI5 mutants of *M. truncatula* and pea were found to be tolerant to desiccation but revealed reduction in seed longevity to an extent of 40–60%. In addition, both the TFs were proposed to be involved in chlorophyll degradation that takes place during the termination of seed maturation process (Zinsmeister et al., 2016; Lima et al., 2017; Smolikova et al., 2017; Lepiniec et al., 2018). In *M. truncatula*, ABI5 has been identified to control gene modules associated to RFO metabolism through induction of seed imbibition protein 1 (SIP1), synthesis of LEA proteins and down-regulation of PhANGs (Zinsmeister et al., 2016). Characterization of ABI5 mutants in pea provided direct evidence confirming the role of ABI5 in regulating seed de-greening process, accumulation of RFOs and acquisition of seed longevity (Zinsmeister et al., 2016). The three-dimensional structure of ABI5 was obtained by homology modeling (Swiss model) and is presented in Figure 3B. The amino acid residues identified in the active sites of ABI5 were identified to be Arg298, His301, His302, Lys305, Asn306, and Ser309 with the help of bioinformatics tool based on Swiss model.

Seed dormancy and longevity are the two important traits that are acquired in the same maternal environment during seed maturation and they ought to share few genetic and developmental mechanisms (Finkelstein et al., 2008; Bewley et al., 2012). However, in *M. truncatula*, Verdier et al. (2013) dissected the maturation events from the end of seed filling to final maturation drying and revealed distinct co-expression modules related to the acquisition of desiccation tolerance and longevity. The acquisition of both desiccation tolerance and dormancy modules was associated with abiotic stress response genes, including LEA genes and the longevity module was enriched in genes involved in RNA processing and translation. Longevity genes were highly connected to two apetala2/ ethylene response element binding protein (*Mt*AP2/EREBP) and two bZip TFs as major hubs (Verdier et al., 2013).

Of late, few common factors are ought to be involved in regulation of initiation and termination of dormancy along with enhancing seed maturation and desiccation processes, that include DOG1 proteins and ABA (Chahtane et al., 2017; Nonogaki, 2019; Smolikova et al., 2020). Hence, understanding of the activities and regulatory control of these key factors would facilitate better understanding of seed longevity. Few studies demonstrated that expression of DOG1 *via* ABA signaling is critical for achieving complete seed maturation (Bentsink et al., 2006; Dekkers et al., 2016; Nonogaki, 2019). In *Arabidopsis thaliana*, five QTLs affecting seed longevity i.e., germination ability after storage (GAAS1-5) were reported to be co-localized with the DOG locus controlling dormancy (Nguyen et al., 2012). However, the study reported a negative association between the seed dormancy and longevity delineating that, seeds are capable of extending their viability either by dormancy or by longevity. Nguyen and Bentsink (2015) hypothesized that trade-off between seed dormancy and longevity could arise due to selective pressure of one on the other that needs to be further investigated. Recently, Carrillo-Barral et al. (2020) proposed that QTLs identified for longevity and dormancy may not always necessarily co-locate and natural variations for these two characteristics might be regulated by different genetic mechanisms. In contrast to this, Dekkers et al. (2016) from their study, found that DOG1 mutants had reduced viability and the knock-down expression of the gene could affect the synthesis of LEA, RFOs and HSPs by interacting with ABI3. Further, DOG1 has been anticipated to act as a positive regulator of ABI5, and in turn activate several LEA and HSP genes (Dekkers et al., 2016). The 3D structure of the protein DOG1 is drawn with the help of Swiss model and presented in Figure 3C. The active sites of DOG1 protein were predicted to be made of Phe101, Leu104, Tyr15, Trp18, Met19, Gln22, Ile55, Phe59, Tyr62, Arg66, Tyr77, Asn83, Glu87, Leu90, Trp92, Met93, Gly94, Gly95, Cys96, and Pro98 residues.

In addition to ABA, auxins were also reported to be involved in regulation of seed longevity (Carranco et al., 2010; Pellizzaro et al., 2020). Transient assays performed in the immature sunflower embryos revealed that, indole acetic acid (IAA) protein *Ha*IAA27 could repress the transcriptional activation and synthesis of *Ha*HSFA9. Whereas, in maturing seeds auxins might enhance seed longevity by destabilizing *Ha*IAA27 protein and enhancing HSFA9 expression (Carranco et al., 2010). Another study revealed involvement of auxin in the induction of the expression of ABI3 and its LEA protein target i.e., early methione1 (EM1), wherein the activity of ABI3 was found to be deregulated in the auxin biosynthesis mutants (Liu et al., 2013; Pellizzaro et al., 2020). Expression of homologs of EM gene in *M. truncatula* has been correlated to be involved in acquisition of either desiccation tolerance or longevity (Chatelain et al., 2012), that implies auxins could have a pronounced role in regulating seed longevity of legumes.

### Implication of Seed Coat Components in Maintenance of Longevity

Seed coat is the means through which the embryo networks with the environment. Thus, seed longevity is mainly affected by composition and structure of seed coat. Several macromolecules are found, whose properties impart physical and chemical resistance to the cells, such as flavonoids (proanthocyanidins), polysaccharides, suberin, cutin, lignin, and lignan. Among these molecules, genes involved in regulating polyphenols especially flavonoids were discussed by few researchers. But the potential candidate genes involved in controlling the activity of other macromolecules remain elusive.

PAs are polymeric flavonoids that accumulate in vacuoles as colorless compounds during early seed development, and encoded by the gene transparent testa 10 (TT10); (Pourcel et al., 2005). Several lines of evidence suggest that, secondary thickening of sub-epidermal cell layer by polyphenols such as flavonoids could have pronounced effect on seed longevity. Thus, the loss of function in Arabidopsis mutants *viz*., TT10 and aberrant testa shape (ATS) (that is required for proper development of all the five layers of testa) exhibited delayed seed coat browning phenotype and germination, respectively. This in turn, resulted in the concomitant reduction in seed longevity under both long-term ambient storage and controlled deterioration conditions (Debeaujon et al., 2000; Clerkx et al., 2003; Pourcel et al., 2005). Flavonoids are transported by multidrug and toxic compound extrusion (MATE) domain containing proteins. The MATE proteins of Arabidopsis and *Medicago truncatula viz*., TT12 and MATE1 were identified to be involved in the biosynthesis of PAs, and mutants of the gene TT12 displayed reduced dormancy phenotype in Arabidopsis (Zhao and Dixon, 2009).

In legumes, during the process of seed desiccation, flavonoids tend to accumulate in seed coat that are oxidized in the presence of polyphenol oxidase or peroxidase, leading to seed coat browning and impermeable to water and oxygen. The reduction in seed coat permeability mediated by flavonoids could limit imbibition damage resulting from solute leakage and enhance seed longevity (Rajjou et al., 2004). Another gene *Gm*Hs1-1, encoding a calcineurin-like protein expressed in the seed coat of soybean is known to control the seed coat permeability. Expression of this gene could enhance calcium concentration and control hard-seededness in soybean, that could enhance longevity with slight reduction in the germination (Sun et al., 2015).

## Conclusion and Way Forward

**Figure 4:**
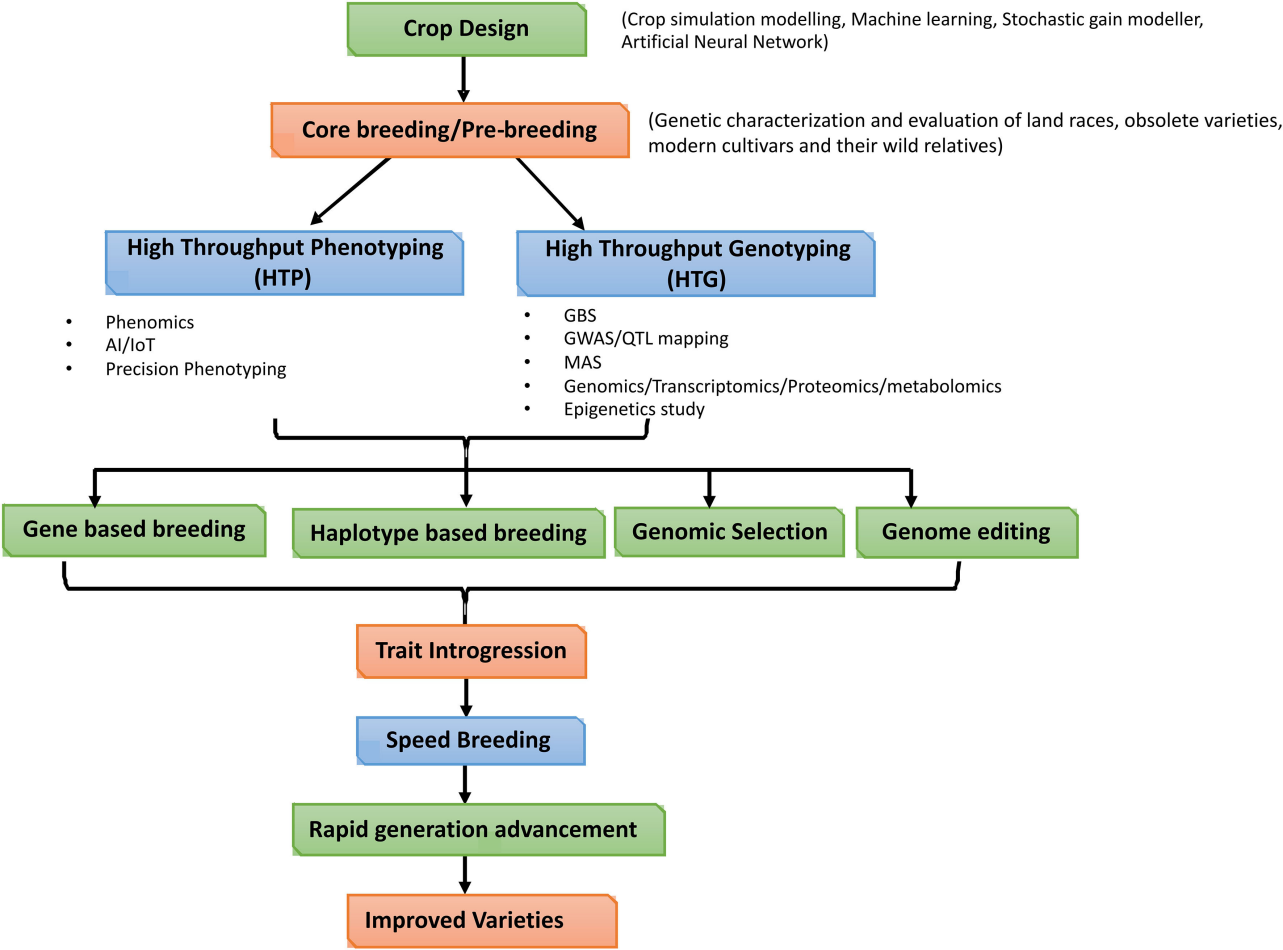
A hypothetical integrated fast forward breeding pipeline AI: artificial intelligence, IoT: internet of things, GBS: genotyping by sequencing, GWAS: genome wide association study, QTL: quantitative trait loci.

Seed longevity is a complex phenomenon as compared to other seed quality traits with preponderant influence on germination. Generally, seed germination potential declines significantly during storage, and seed longevity is a key determinant of crop production. In legumes and crop species that store oil reserves inside the seed, safeguarding and conservation of viability during storage is strenuous. Several studies deciphered different extrinsic factors such as humidity, temperature and oxygen concentration as critical seed longevity determinants. In addition to external factors, several intrinsic factors play a key role in dictating the germination potential of seeds, based on gene and gene product interactions as well as complex molecular networks. Hence, understanding the genetic and molecular mechanisms involved in regulation of seed longevity and mechanisms governing genome maintenance in the seeds, through advanced omics-based approaches is critically essential. Understanding the mechanistic basis of seed longevity stands as a prerequisite and directly assists breeding programmes in the development of varieties with enhanced seed longevity and vigor, as well as strengthening the conservation of genetic resources in the seed banks. Besides, the knowledge of protein's 3D structure will be useful in understanding how the protein works, and use that information for modification of genes/protein associated with seed longevity and its associated seed quality parameters. Apart from this, epigenetic regulation is a heritable-change that occurs at DNA, RNA and histone level, where modification in the chromatin structure is the basis for gene regulation. It is assumed that dramatic epigenetic mechanism especially DNA methylation play a significant role in desiccation tolerance both in orthodox and recalcitrant seeds at the late seed maturation stage (Plitta-Michalak et al., 2018; Smolikova et al., 2021). Storage and aging influenced epigenetic stability consequently alter DNA methylation state of seeds and seedlings. This DNA methylation reprogramming activates transposable elements and could be involved in cytogenetic instability through heterochromatin modifications (Pirredda et al., 2020). Therefore, there is a need to dig deep into the epigenetic regulation of gene expression that could identify potential targets for integrating into selective breeding programs such as genomics assisted breeding, haplotype-based breeding, chromosome, specific breeding etc. Investigating the seed longevity with the help of a hypothetical integrated fast forward breeding pipeline (Figure 4) in accordance with other associated seed quality parameters such as germination, dormancy and vigor aspects is the road ahead for sustainable development and successful crop enterprise systems.

## Author Contributions

VR and SC: writing—original draft and review and editing. SuK: writing—review. SV, SS, UK, and BK: writing. SaK, ANS, and HVS: review, supervision, editing and visualization. All authors contributed to the article and approved the submitted version.

## Conflict of Interest

The authors declare that the research was conducted in the absence of any commercial or financial relationships that could be construed as a potential conflict of interest.

## Publisher's Note

All claims expressed in this article are solely those of the authors and do not necessarily represent those of their affiliated organizations, or those of the publisher, the editors and the reviewers. Any product that may be evaluated in this article, or claim that may be made by its manufacturer, is not guaranteed or endorsed by the publisher.
